# Supplementary material for: Short-term prophylaxis in patients with angioedema due to C1-inhibitor deficiency undergoing dental procedures: An observational study
Source: PLoS One. 2020 Mar 12;15(3):e0230128. doi: 10.1371/journal.pone.0230128 (PMC7067439; doi:10.1371/journal.pone.0230128)
Supplement: Attachment — Submitted filename: Response to Reviewers.docx [file pone.0230128.s002.docx]

Response to Reviewers

Reviewer #1: I think this paper can only be accepted if the author's add and answer the following points in the current submission. It needs more statistical analysis to be done with statistical software packeges.
1. Kindly add co-relation of patient age with the disease pattern. In current article patients age 8-85 years are added, which is a wide range.

2. Add some more statistical data analysis as well as some statistical graph in this article.

As required by Reviewer #1, we performed more statistical analyses. We investigated the possible correlation between age and oral disease severity and between age and angioedema severity. We added two paragraphs and one figure in the Results section.

3. Add some more discussion to correlate the immunological effect of the disease process as well as therapeutic approach.

4. Kindly add some more current relevant references.

We added a more thorough explanation about the relationship between infections and occurrence of angioedema attacks in the Discussion section and we circumstantiated these data with appropriate bibliographic references.

5. Add some pictures to show the Angioderama and clarify the scoring process with more supporting images in the current population of the patient.

We showed our scoring systems, adding Table 1 and Table 2 in the Methods section (see “Severity of oral and angioedema disease” paragraph).

Reviewer #2: The authors present data from a study on the management of dental procedures in patients with HAE or AAE due to C1-Inhibitor deficiency. It is adequately written and the conclusions presented are appropriately supported by the data. Although most of the conclusions reached by the authors are not novel, they do provide experimental support to previously reported observations with important implications for the management of these patients. Because of dealing with a rare pathology, the relatively small number of individuals recruited for this study is not to be considered a substantial limitation in its design.

We thank the Reviewer for appreciating this work.

Minor points:

- Lines 175-176: The manuscript states that “….Most patients recruited in this study had a moderate-to-high score for the severity of oral pathology”. However, according to Table 1, most of the patients studied (20/27) had a Mild to Moderate phenotype.

The intended meaning was that the sum of patients with moderate and severe oral pathology is greater than 50%. In order to avoid misunderstandings, we changed the sentence indicating the precise percentage (59.25%).

- Lines 179-180: The amelioration of the patients’ HAE course after dental care is a reasonable conclusion. Though, significant improvement of HAE was observed in as little as 20% of the studied cases. Wouldn’t it be expectable to find a general improvement in the cohort after dental management? Were these 4 patients showing amelioration of HAE those exhibiting worse oral pathological scores?; that is, is there any correlation between the oral disease score and the beneficial effect of dental care on HAE?
Can the authors speculate in the discussion section on the hypothetical causes of the lack of response in an 80% of cases?

We thank the Reviewer, as our manuscript would certainly benefit from further deepening of this topic.

We eliminated a possible trigger of angioedema attack, but, as it not possible to eradicate the underlying pathology, we did not expect an improvement in all the patients. No one worsened and 4 improved: we added in the Discussion section a thorough analysis of the severity of oral pathology.

The average oral pathology score was slightly worse in the subpopulation who experienced an amelioration compared with the other patients (4.75 vs 4). This finding indicates that the subpopulation who benefits most from dental procedures has more severe oral pathology.

- Line 223: Previous studies have also shown that pdC1INH prophylaxis is less effective for AAE-C1INH as compared to HAE-C1INH (reviewed for example in Cicardi et al, 2014). This observation is coherent with the increased C1INH catabolism characteristic of AAE-C1INH patients and may be influenced by the presence or absence of anti-C1INH autoantibodies in the patient, as suggested by the authors’ data.
However, most experts recommend antifibrinolytic agents for the prophylaxis of AAE-C1INH; why were the two AAE-C1INH patients receiving pdC1INH instead? The reason of this treatment choice should be clear in the manuscript.

We thank the Reviewer for this comment. There is no evidence from randomized clinical trials that antifibrinolytic agents are effective for STP in AAE-C1INH. These agents are contraindicated in some categories of patient, such as those receiving antiplatelet or anticoagulant therapy and those with procoagulant condition. This was the case of our patients and the reason why we chose pdC1INH for STP.

We clarified the reason of the treatment choice in the manuscript in the Discussion section.
